# Association between allergic rhinitis and asthma in a Northern Alberta cohort

**DOI:** 10.1186/1916-0216-42-58

**Published:** 2013-12-19

**Authors:** Caroline C Jeffery, Mohit Bhutani, Harissios Vliagoftis, Erin D Wright, Hadi Seikaly, David WJ Côté

**Affiliations:** 1 Division of Otolaryngology-Head and Neck Surgery, Department of Surgery, University of Alberta, 8440 112 Street, Edmonton, Alberta T6G 2B7, Canada; 2 Division of Pulmonary Medicine, Department of Medicine, University of Alberta, 8440 112 Street, Edmonton, Alberta T6G 2B7, Canada

## Abstract

**Background:**

Many published epidemiologic studies confirm a marked increase in the prevalence of asthma and allergic rhinitis. The link between allergic rhinitis and asthma has been extensively studied and approximately 75% of patients with asthma have allergic rhinitis. The proportion of patients with asthma in populations of allergic rhinitis patients has not been well studied.

**Objective:**

The purpose of this study is to estimate the prevalence of undiagnosed asthma in a specific population of patients presenting to an Otolaryngologist with symptoms of allergic rhinitis.

**Study design:**

Prospective cohort study. Methods: Patients presenting with symptoms of allergic rhinitis to two tertiary care Rhinology practices in Northern Alberta were asked to undergo allergy skin testing, serum IgE quantification, and pulmonary functional testing. Patients with previous asthma screening or known history of reactive airway disease or asthma were excluded.

**Results:**

107 patients with allergic rhinitis symptoms were recruited between September 2010 to January 2013. Patients predominantly had perennial or persistent rhinitis (64.5%) with moderate-severe symptoms (50.5%). While only 14.9% of patients had abnormal IgE levels, 68.8% had positive skin testing. Abnormal pulmonary function tests were obtained in 39.1% of patients and 26.1% of patients were diagnosed with asthma.

**Conclusions:**

There is a high prevalence of undiagnosed asthma in patients presenting to tertiary Rhinology care with moderate to severe allergic rhinitis symptoms. Screening lung function testing should be considered in this patient population.

## Introduction

Many published epidemiologic studies examining populations in Europe over the last few decades have confirmed a marked increase in the prevalence of asthma and allergic rhinitis [1-3]. One recently published study estimates that the overall prevalence of allergic rhinitis in the Canadian population is 20% with a high burden of disease, including limitations on lifestyle and poor symptom control [4]. Similarly, overall prevalence of asthma remains high in Canada. Direct costs of managing this chronic condition is increasing and it remains the number one cause of decreased productivity and missed work days [5]. The link between allergic rhinitis and asthma has been extensively studied [6]. Approximately 75% of patients with asthma have allergic rhinitis [7]. The proportion of patients with asthma in populations of allergic rhinitis patients has not been well studied, with small studies reporting ranges between 10 to 40% and no studies looking at this proportion in the Canadian populations [7-9]. Studies have also shown that allergic rhinitis has a major impact on asthma morbidity and that treating allergic rhinitis may also impact asthma control [10].

Several explanations for this close relationship prevail in the literature. At the forefront is the unified airway hypothesis, which states that both upper and lower airway allergic symptoms are manifestations of the same atopic disease [11]. Asthma and rhinitis share many pathophysiologic characteristics [12] and studies suggest that subclinical allergic inflammation is present in the lower airway of patients with allergic rhinitis [13]. Clinically, both allergic rhinitis and asthma are triggered by many of the same environmental allergens [14]. Furthermore, it is proposed that a loss of nasal function, presence of a nasobronchial reflex, and propagation of inflammation from the upper to the lower airways also contribute to combined nasal and airway inflammation [15].

The current understanding of shared pathophysiology between allergic rhinitis and asthma prompted the World Health Organization’s ARIA (Allergic Rhinitis and its Impact on Asthma) to recommend asthma screening in patients with allergic rhinitis. Nonetheless, it is not current practice by most Otolaryngology-Rhinology practitioners in Canada to order this screening routinely for patients with symptoms consistent with allergic rhinitis. The purpose of this cross-sectional study is to estimate the prevalence of undiagnosed asthma in a specific population of patients presenting to an Otolaryngologist with symptoms of allergic rhinitis.

## Methods

University of Alberta Research Ethics Board approval was obtained prior to commencement of study (Pro00032514). Adult patients (age ≥ 17 years of age) presenting to two Academic Otolaryngologists with rhinology practices at the University of Alberta with one or more symptoms of rhinitis were prospectively recruited between September 2010 and January 2013. Patients were diagnosed with allergic rhinitis if at least one symptom of nasal itching, sneezing, nasal obstruction, congestion, rhinorrhea, and/or hyposmia was present in association allergen exposure. Patients with concomitant diagnosis of chronic rhinosinusitis with or without polyposis were not excluded. Patients with known or previously diagnosed allergic asthma or reactive airway disease were excluded.

Severities of nasal symptoms were categorized as intermittent versus persistent and mild versus moderate-severe in accordance with the WHO Allergic Rhinitis and its Impact on Asthma 2008 Guidelines [http://www.whiar.org/Documents&Resources.php]. A four-point asthma-screening tool was also administered to each patient (Table 1). Recruited patients subsequently underwent skin-prick specific IgE allergy testing, total serum IgE quantification, and pulmonary function testing. Diagnosis of asthma was based on demonstration of reversible airway obstruction with bronchodilator administration as indicated by ≥12% increase in FVC (forced vital capacity) or FEV1 (forced expiratory volume in one second). Severity of asthma on pulmonary function testing was classified by FEV1/FVC ratio according to standard ranges (http://www.ginasthma.org/). All data recorded were compared to norms based on age, gender, race, weight and height. All patients underwent testing at the same diagnostic laboratory. Results of testing were prospectively collected in a database.

**Table 1:** Demographics of study patients

		**Number of Patients**	**% of Total**
**Average Age**		39.8 ± 14.4	
**Sex**	Male		50.5%
**Classification of AR**	Seasonal AR	39	36.4%
	Perennial AR	64	59.8%
	Intermittent	38	51.4%
	Persistent	69	64.5%
	Mild Symptoms	53	49.5%
	Moderate-Severe Symptoms	54	50.5%
**Rhinologic History**	CRSwP	19	17.8%
	Prior Sinonasal Surgery	7	6.5%
	ASA Insensitivity	5	4.7%
	Recent oral corticosteroid	3	2.8%
**Atopic History**	Family history of Atopy	46	43.0%
	Personal History of Atopic Dermatitis	24	22.4%
	Patient reports environmental allergies	84	78.5%

### Risk factor analysis

SPSS 20.0 was used for statistical analysis. Factors were examined for association with increased likelihood of asthma using Mantel-Haenszel tests followed by regression analysis using likelihood ratio tests. A significance level of p < 0.05 was used for all analysis.

## Results

**Figure 1:**
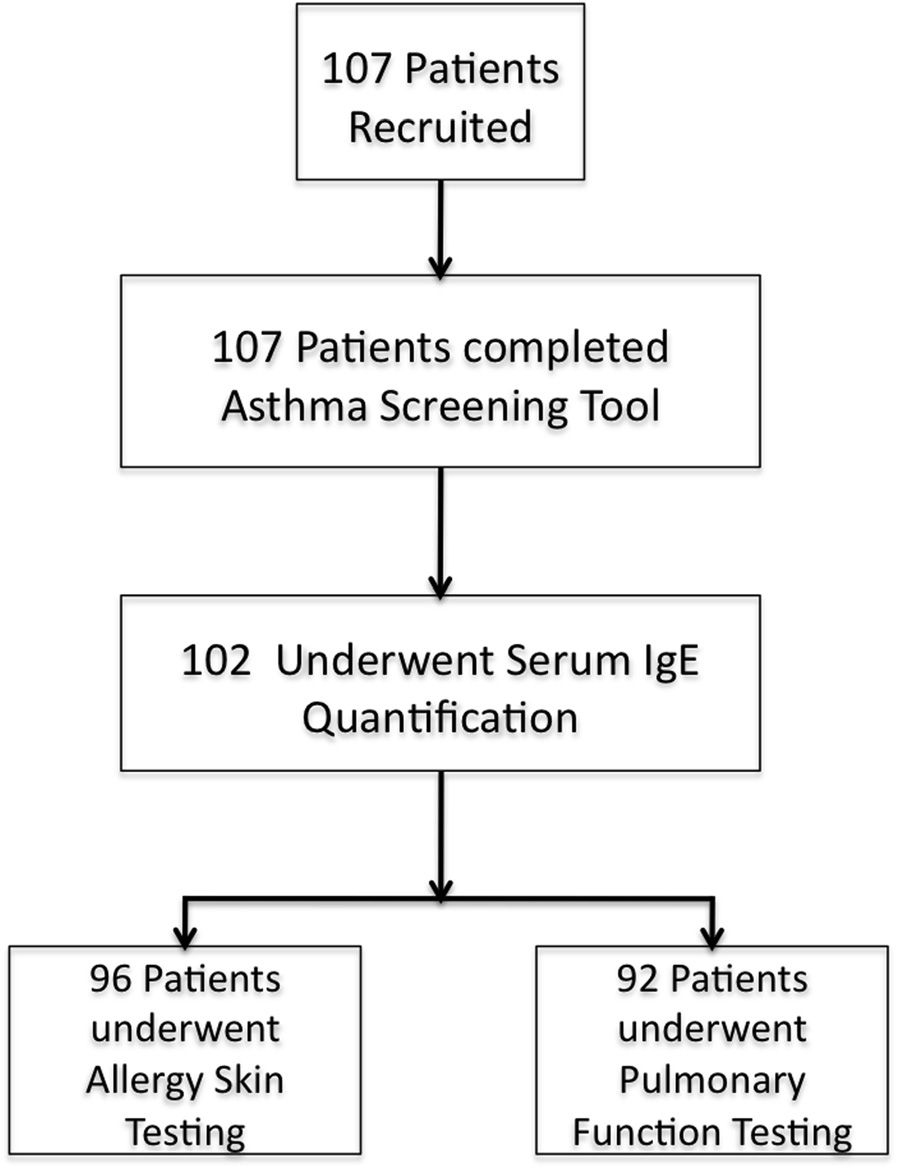
Patient recruitment and attrition.

**Figure 2:**
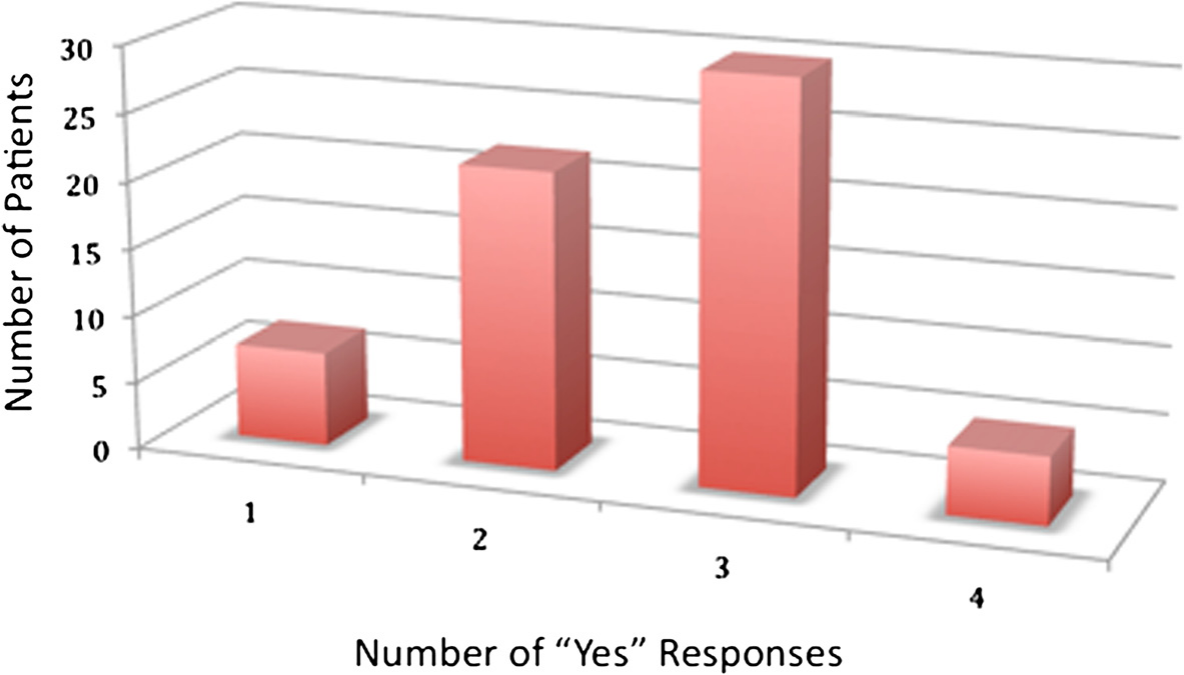
Distribution of responses to asthma screening tool.

One hundred and seven patients were recruited during the study period (Figure 1 and Table 1). The majority of patients had persistent and moderate-severe symptoms of allergic rhinitis (Table 1) and 17.8% of patients had clinical and/or radiographic evidence of chronic rhinosinusitis with or without polyposis. Almost 87% of patients responded “yes” to two or more questions from the *Asthma Screening Tool* (Figure 2).

### IgE-allergy skin testing

68.8% of patients had positive IgE-specific skin testing (Table 2). On average, patients were sensitized to 3.5 allergens (Range 1 to 20). 20.0% of patients had negative skin-prick testing despite a history of environmental allergies.

**Table 2:** Results of tests of sensitization and aeroallergy

		**Number of patients (%)**
Underwent Total Serum IgE		102 (100 %)
	Abnormal Total Serum IgE	16 (15.7 %)
Underwent Skin-Prick Testing		96 (100 %)
	Positive Skin-prick Testing	66 (68.8 %)
	Average No. of Allergens Sensitized	3.5 ± 2.4

### Pulmonary function testing

92 patients underwent pulmonary function testing with abnormalities found in 36 patients (39.1%). Of the abnormal results, 24 patients had a diagnosis of asthma (26.1%) based on diagnostic criteria outlined in ‘Methods’. The distribution of severity of asthma is shown in Table 3.

**Table 3:** Results of pulmonary function testing

			**Number of patients (%)**
Underwent PFT			92 (100%)
Abnormal PFT			36 (39.1)
	Asthma		24 (26.1%)
		*Mild*	22 (23.9%)
		*Moderate*	2 (2.2%)
		*Severe*	0 (0.0%)
	Restrictive lung disease or chest wall abnormality		5 (5.4%)
	Diffusion abnormalities		2 (2.2%)
	Other		5 (5.2%)

### Risk factor analysis

Regression analysis failed to show any independent risk factors for increased risk of asthma diagnosis (Table 4).

**Table 4:** Mantel-Hanzel odds ratio estimates of various risk factors for asthma on pulmonary function testing

**Risk factor**		**Odds ratio**	**95% CI**	**P-value**
Male Sex		1.711	0.667 – 4.376	0.263
Type of AR	Seasonal	0.758	0.492 – 4.076	0.573
	Perennial	0.875	0.290 – 1.984	0.779
	Intermittent	1.296	0.345 – 2.220	0.596
	Persistent	0.786	0.500 – 3.360	0.632
	Mild	1.726	0.303 – 2.040	0.252
	Moderate-Severe	0.627	0.678 – 4.396	0.324
Sinonasal Disease	CRS	0.645	0.238 – 1.584	0.387
	Recurrent ABRS	1.121	0.111 – 11.326	0.923
	CRS with Polyposis	0.900	0.266 – 1.944	0.866
3 or More “Yes” to Asthma Sreening tool		1.191	0.465 – 3.049	0.480
Atopic History	Family History	0.759	0.296 – 1.944	0.565
	Atopic Dermatitis	0.656	0.198 – 2.168	0.489
	Subjective Allergies	1.013	0.328 – 3.134	0.982
Positive Skin Testing		1.417	0.492 – 4.076	0.518
Abnormal IgE		2.667	0.735 – 9.669	0.136

## Discussion

This study found a high rate of undiagnosed asthma in a population of patients with allergic rhinitis presenting to tertiary care Rhinology in Northern Alberta. The results of this study support the existing evidence for a strong association between asthma and allergic rhinitis. It also supports studies that have demonstrated AR as an independent risk factor for asthma [7].

To our knowledge, there are currently no other studies in the literature that examine the rate of undiagnosed asthma in allergic rhinitis patients. Also, no studies have examined, specifically, the value of asthma screening in this population despite recent ARIA guidelines, which recommended that persistent allergic rhinitis should be evaluated for asthma [16]. We found that 26.1% of patients in our cohort had asthma on pulmonary function testing. Majority of patients in our cohort were subsequently referred to Pulmonary Medicine for further management of their lower-airway disease. Although not all patients required immediate initiation of medical management, earlier detection of asthma potentially facilitates patient counseling and long-term management of their disease. In addition, substantial literature exists that demonstrate benefits of managing AR in patients with co-morbid asthma [17].

Our study failed to identify any specific risk factors for abnormal pulmonary function test results in patients with allergic rhinitis. Specifically, a family history of atopy, positive allergy skin testing and answers to the 4-point allergy-screening tool failed to predict likelihood of asthma. Some authors have suggested that a serum IgE level > 140 IU/ml may be predictive of atopic disease [18], while others have suggested lower limits or even limited utility [19], [20]. At our institution, a total IgE of greater than 120 IU/mL is considered abnormal, but this finding in our patients, while yielding an Odds Ratio of 2.667 with findings of asthma on PFT, failed to reach statistical significance. Further studies are necessary to examine other potential risk factors for increased likelihood asthma in AR patients such as duration of allergic rhinitis, geography, and respiratory sensitizer exposure.

Admittedly, this is a cross-sectional study with a relatively small sampling of patients in Alberta and is highly selected -- the patients in our study had significant nasal complaints as demonstrated by a high proportion of patients with moderate-severe (50.5%) and persistent rhinitis (64.5%). This is in contrast to the mild symptoms seen in the general population who are likely managed by primary care practitioners [21]. In addition, the study patients would benefit from observation for longer periods of time. It is possible that patients with initially normal PFTs may subsequently develop evidence of lower airway disease if followed over time. Finally, the overall prevalence of asthma in Canada varies between 5 and 10%, with Alberta reporting the highest rates in Canada of up to 10.2% [22], [23]. Thus, the results of our study may not be applicable to other regions. Similar studies from other centers in Canada would help clarify this question.

Despite these limitations, the results of this study remain useful to clinicians that treat patients with a spectrum of AR severity. It reinforces the fact that AR is not a trivial disease and should be managed aggressively to optimize progression of airway reactivity. In patients with persistent and severe disease, there is a high rate of undiagnosed, but clinically significant asthma and we feel this warrants consideration for increased asthma screening by practitioners who see allergic rhinitis patients. Future studies examining the cost-effectiveness of asthma screening in this population are necessary before widespread adoption.

## Conclusions

There is a high prevalence of undiagnosed asthma in patients presenting to tertiary Rhinology care with moderate to severe allergic rhinitis symptoms. Screening lung function testing may be considered in this patient population.

## Competing interests

The authors declare that they have no competing interests.

## Authors’ contributions

DWJC, EDW, MB, and HV conceived and designed the study. DWJC, EDW, MB, HV, and CCJ collected the study data. CCJ analyzed the data and prepared the manuscript. DWJC, EDW, MB, HV, HS, and CCJ approved the final manuscript.
